# Supplementary material for: Synthesis of Bioactive Silver Nanoparticles Using New Bacterial Strains from an Antarctic Consortium
Source: Mar Drugs. 2022 Aug 31;20(9):558. doi: 10.3390/md20090558 (PMC9505403; doi:10.3390/md20090558)
Supplement: Supplementary file 1 [file marinedrugs-20-00558-s001.zip › marinedrugs-1842489-supplementary.pdf]

Supplementary Material

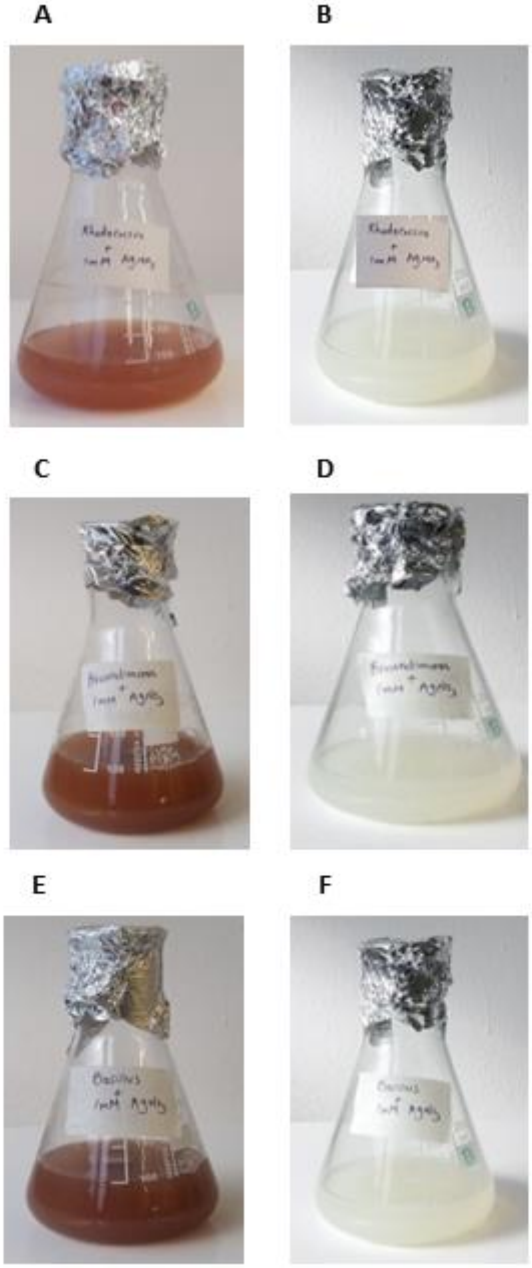

Figure S1. AgNP synthesis. Visual observation of the synthesis by medium color change from colorless to dark brown during 24 hours of incubation of *Rhodococcus* (A), *Brevundimonas* (C), and *Bacillus* (E) with 1mM $AgNO_3$.: Flasks with 1mM $AgNO_3$ containing heat killed *Rhodococcus*, *Brevundimonas,* and *Bacillus* are shown in B, D, and F, respectively.

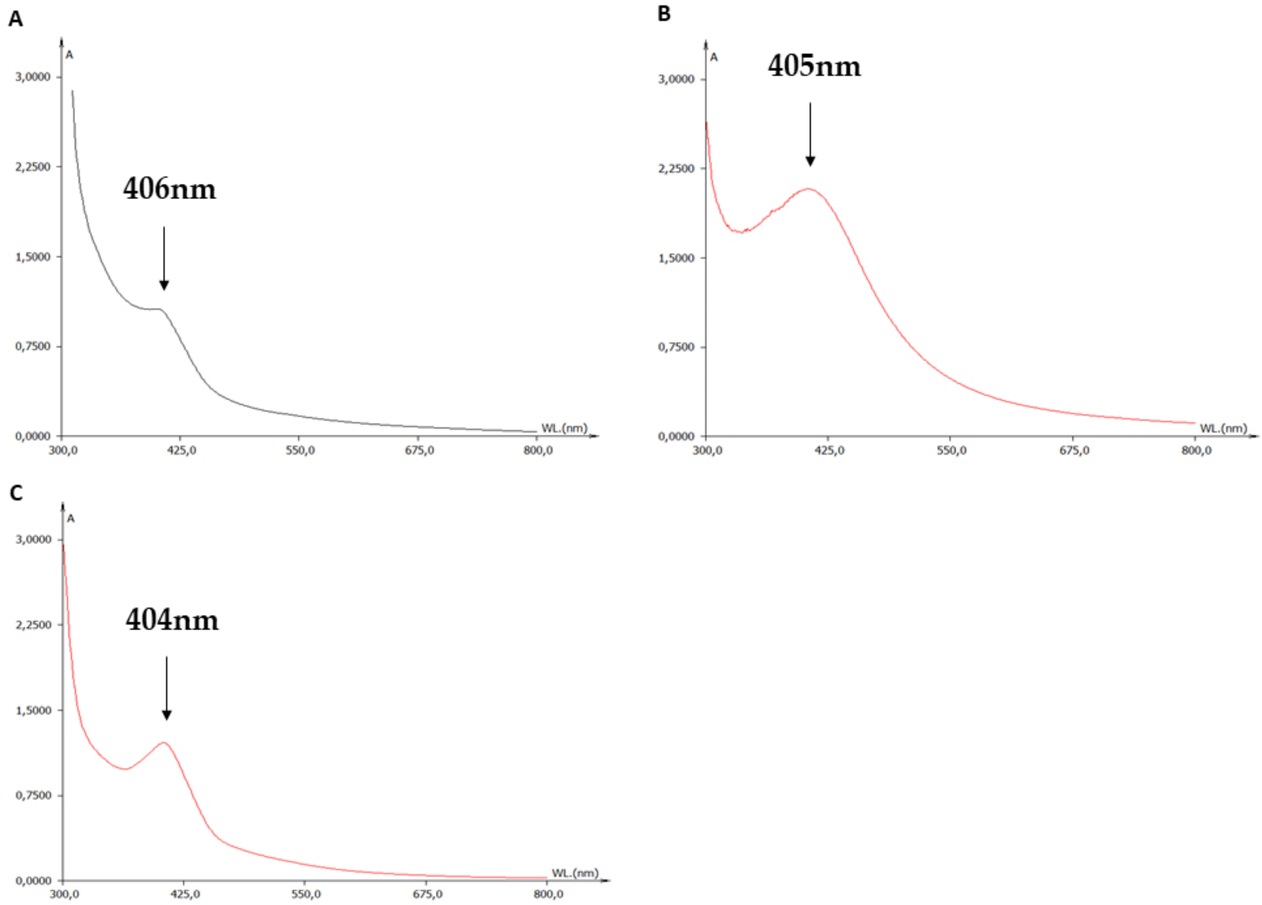

Figure S2. UV−Vis absorbance spectra of AgNPs produced by ***Rhodococcus* (A), *Brevundimonas* (B), and *Bacillus* (C)**.

Supplementary Results

1 Fourier-transform infrared spectroscopy (FTIR) analysis

FTIR measurements were carried out to identify possible interactions between silver salts and protein molecules, which could account for the reduction of Ag+ ions and stabilization of AgNPs. The amide linkages between amino acids residues in proteins give rise to the well-known signatures in the infrared region of the electromagnetic spectrum.

Brevundimonas analysis showed the broad intense absorption bands at 3365.34 cm-1, 3261.45 cm-1, and 3056.88 cm-1 attributed to –NH stretch in aromatic amines, primary amines and amides, -OH stretch of alcohols and phenols and =C-H stretch of aromatic and unsaturated hydrocarbons, respectively (**Figure S3A**). The bands shown at 2957.75 cm-1 and 2161.21 cm-1 represent the C–H stretching of aromatic compound of phenol group, and C≡N stretch of Thiocyanates and Isothiocyanates, respectively. The bands at 1580.09 cm-1 and 1515.16 cm-1 represent the $-NH_2$ stretch of primary amines and Ring stretch of triazine compounds,

respectively. The characteristic bands recorded at 1470.48 cm-1 and 1455.02 cm-1 represent the vibrations of aliphatic compounds and -CH_2 scissors vibration, while the band at 1401.73 cm-1 represents -OH bending of carboxylic acids. The distinct band at 1350.31cm-1 indicates NO_2 stretch in aliphatic nitro compounds, while the band observed at 1188.31 cm-1 confirms C-N stretch of aromatic compounds and the band observed at 1079.82 cm-1 confirms the presence of C-O-C stretch of ethers. The bands at 978.25 cm-1 and 921.94 cm-1 indicate CH=CH2 of vinyl compounds and the band at 846.20 cm-1 represents C-N stretch of nitro group.

Rhodococcus analysis (**Figure S3B**) showed the broad intense absorption bands at 3250.71 cm-1 and 3055.82 cm-1 attributed to -OH stretch of alcohols and phenols and –NH stretch in aromatic amines, primary amines and amides. The bands at 2957.67 cm-1 and 2160.97 cm-1 represent the -CH3 and -CH2 stretch of aliphatic compounds and C≡N stretch of thiocyanates and isothiocyanates, respectively. The band at 1579.12 cm-1 and 1515.09 cm-1 are for COO^- stretch of carboxylic acid and ring stretch of triazine compound, respectively. The characteristic bands recorded at 1470.71 cm-1 and 1454.93 cm-1 are for the -CH_2 scissors vibration in aliphatic compounds and the band at 1400.38 cm-1 is for -OH bending of carboxylic acids. The distinct bands found at 1350.32 cm-1, 1189.00 cm-1, and 1078.97 cm-1 indicate -NO_2 in aliphatic nitro compounds, C-N stretch of aromatic compounds, and C-O-C stretch in ethers, respectively. The bands observed at 974.77 cm-1 and 892.25 cm-1 confirm CH=CH2 stretch of vinyl compounds.

Bacillus analysis (**Figure S3C**) showed the broad intense absorption bands at 3042.10 cm-1, attributed to alkenyl -C-H Stretch. The bands at 2957.56 cm-1 and 2161.22 cm-1 represent the -C-H stretch of alkyl compounds. The bands at 1579.47 cm-1 and 1515.19 cm-1 are for -C=C bending in aromatic compounds. The band at 1454.96 cm-1 is for -CH_2 scissors vibration in aliphatic compounds. The characteristic bands recorded at 1399.07cm-1, and 1349.14 cm-1 are for the vibrations of $N0_2$ stretch in aliphatic nitro compounds. The bands observed at 1188.65 cm-1 and 1078.18 cm-1 confirm -C-OH stretch in secondary alcohols. The bands at 975.53 cm-1 and 923.87 cm-1 correspond to P-O-C stretch in alkyl phosphates

A

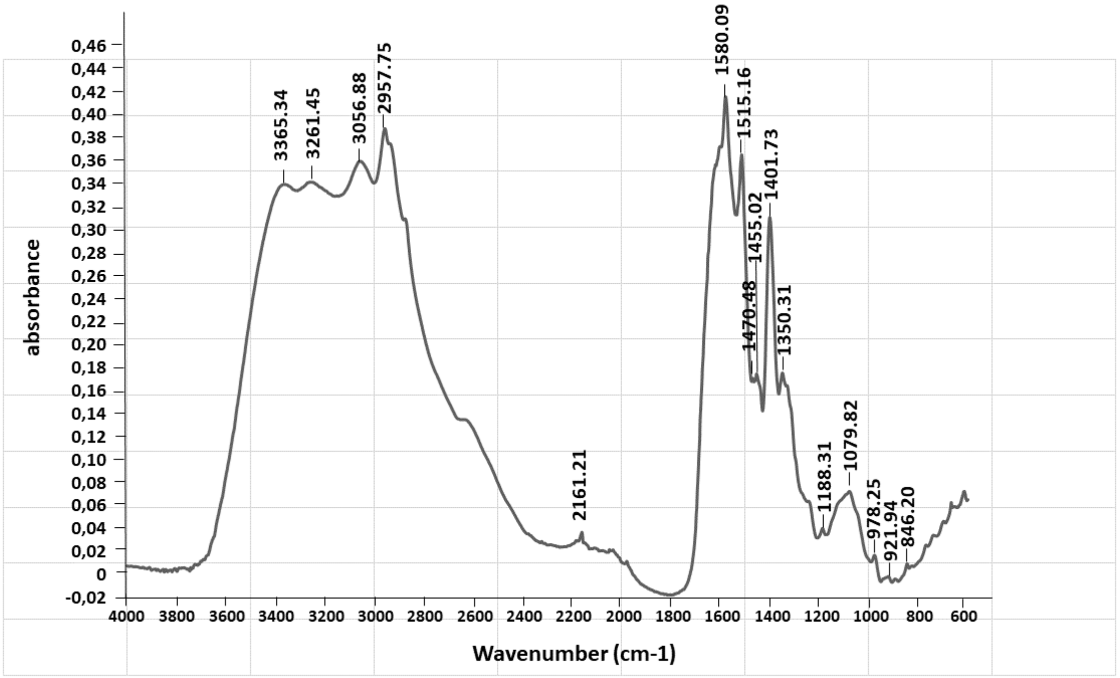
absorbance
Wavenumber (cm-1)
3365.34
3261.45
3056.88
2957.75
2161.21
1580.09
1515.16
1455.02
1401.73
1470.48
1350.31
1188.31
1079.82
978.25
921.94
846.20

B

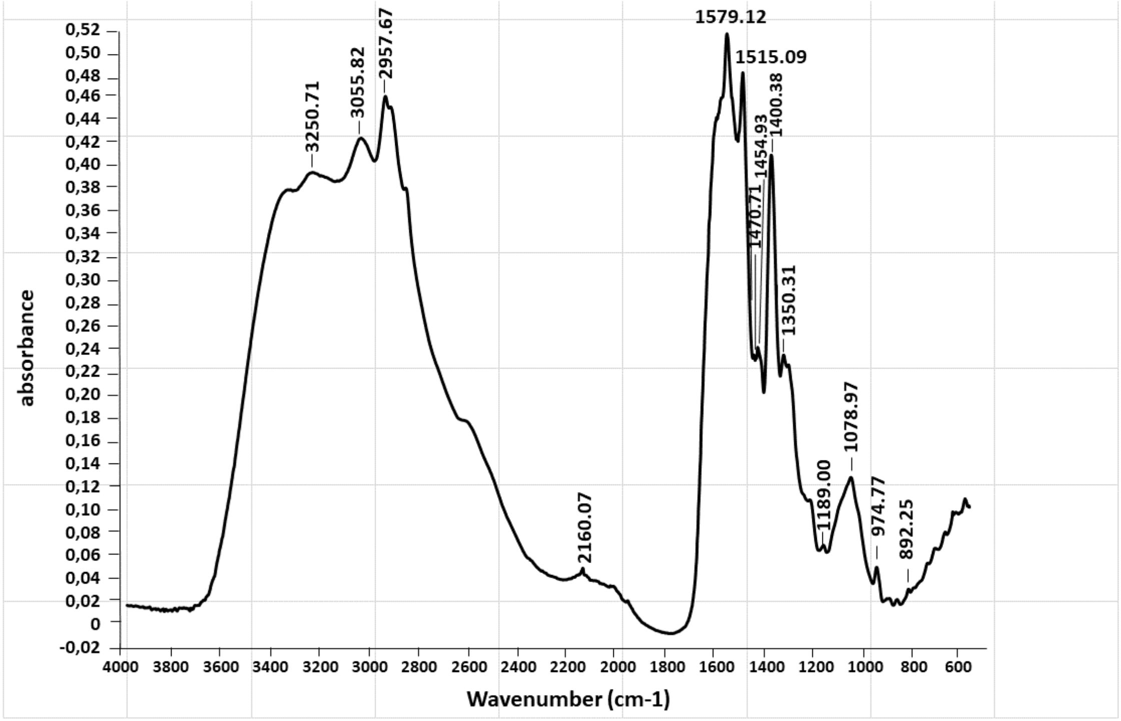
absorbance
Wavenumber (cm-1)
3250.71
3055.82
2957.67
2160.07
1579.12
1515.09
1454.93
1400.38
1470.71
1350.31
1189.00
1078.97
974.77
892.25

C

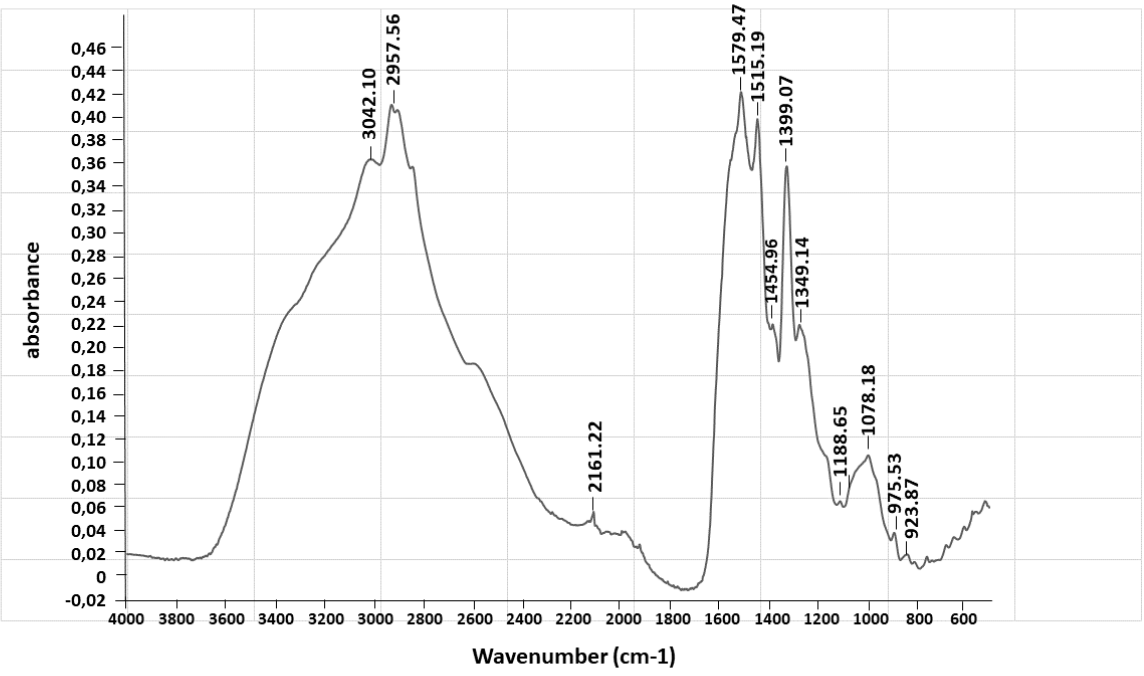

Figure S3. FTIR spectra of AgNPs from *Brevundimonas* (A*), Rhodococcus* (B), *Bacillus* (C). FTIR analysis of the possible interactions between silver salts and protein molecules, which could account for the reduction of Ag+ ions and stabilization of AgNPs

2. Zeta Potential Measurements

Zeta potential measures the charge and the potential stability of the particles in the colloidal solution. AgNPs generally carry a negative charge. The synthesized AgNPs showed negative charge indicating that the sample is stable at room temperature: the zeta potential of *Rhodococcus, Brevundimonas* and *Bacillus* were − 32.8 mV, − 29.6 mV, and − 28.1 mV, respectively (**Figure S4**). The obtained single peak indicated that the quality of the synthesized silver NPs was good.

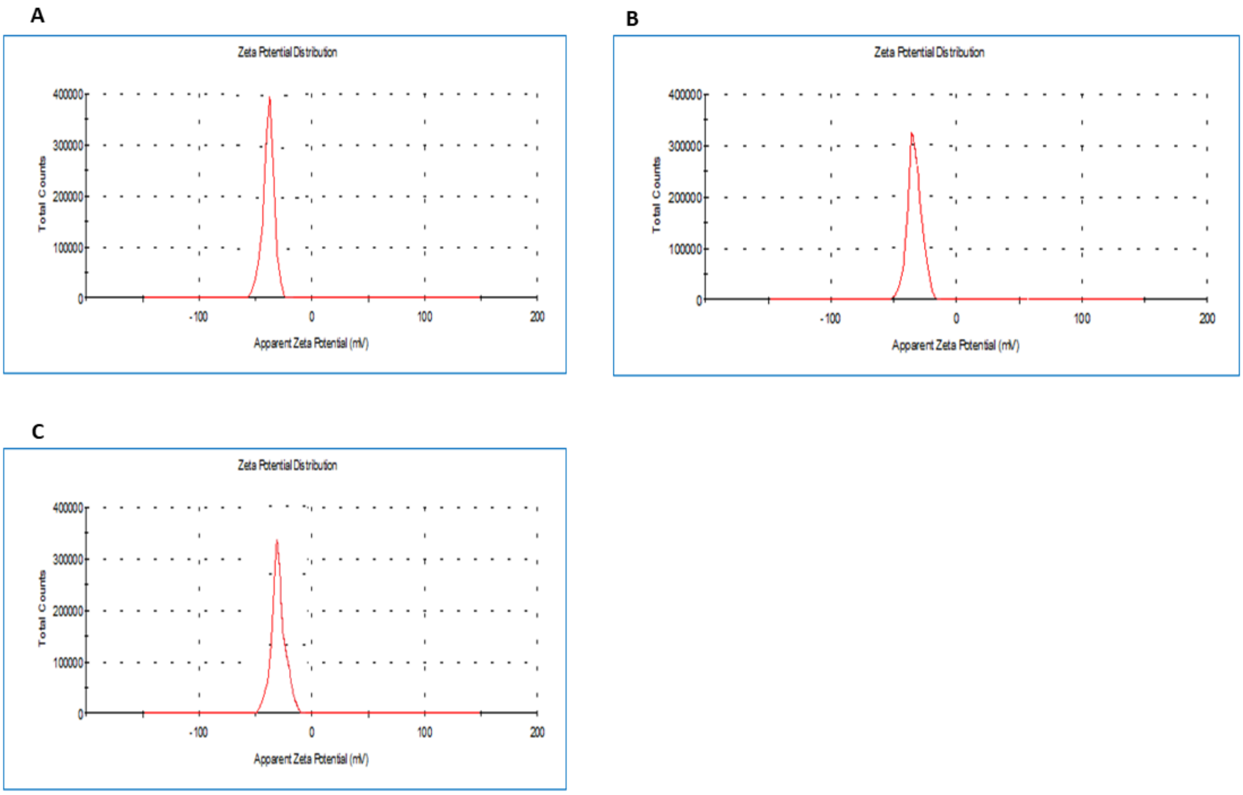

Figure S4. Zeta potential of AgNPs from *Rhodococcus (A), Brevundimonas (B),* and *Bacillus (C).* *Rhodococcus, Brevundimonas,* and *Bacillus* showed zeta potential values of − 32.8 mV, − 29.6 mV, and − 28.1 mV, respectively.

3. X-ray Powder Diffraction (XRD) analysis

The presence of metallic AgNPs was confirmed by XRD analysis (**Figure S5**).

The diffracted intensities for *Rhodococcus* (**Figure S5A**) were recorded from 20° to 80°. Four strong Bragg reflections at 38.45°, 46.35°, 64.75° and 78.05° correspond to the planes of (1 1 1), (2 0 0), (2 2 0) and (3 1 1) respectively, which can be indexed according to the facets of face centered cubic (fcc) crystal structure of silver. Joint Committee on Powder Diffraction Standards (JCPDS) file No. 04-0780.

The crystalline nature of *Bacillus* synthesized AgNPs (**Figure S5B**) is confirmed by XRD. Three major peaks were observed in the XRD spectrum. XRD spectrum shows separate peaks at $2\theta = 38.23°, 44.42°, 64.44°$, and 77.39°, which correspond to (111), (200), (220), and (311), respectively. The XRD spectrum of AgNPs is analyzed with respect to standard AgNPs published in JCPDS file No. 04-0783.

X-ray diffraction (XRD) patterns of AgNPs synthesized from *Brevundimonas* indicate that the structure of AgNPs is the fcc structure of metallic silver (**Figure S5B**). In addition, the diffraction peaks at 2θ values of 38.1, 44.3, 64.4, and 74.3 could be attributed, respectively, to (111), (200), (220), (311) planes of pure silver ions

indicating the biosynthesis of AgNPs. Accordance with the standard metallic silver XRD pattern JCPDS No. 89-3722.

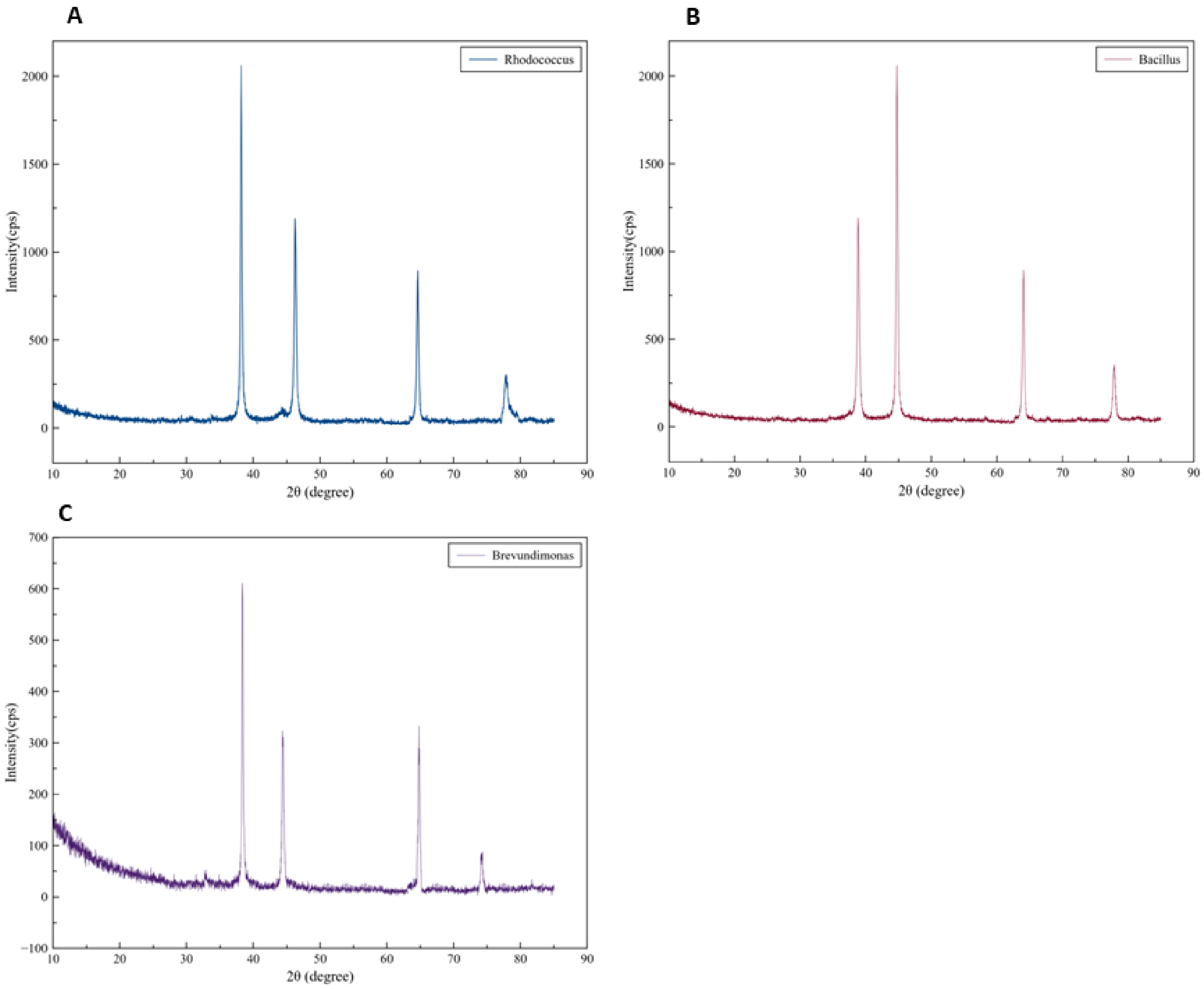

Figure S5 X-ray diffraction profile of AgNPs from *Rhodococcus* (A), *Bacillus* (B) and *Brevundimonas* (C). XRD analysis revealed face centered cubic crystal structure of AgNPs.

4. Scanning electron microscopy

Scanning Electron Microscopy (SEM) was used to determine the size, shape, and distribution of AgNPs. SEM images revealed different sizes of AgNPs from the bacteria ranging from 20 to 70 nm. The NPs are uniform in diameter and show a spherical shape (**Figure S6)**.

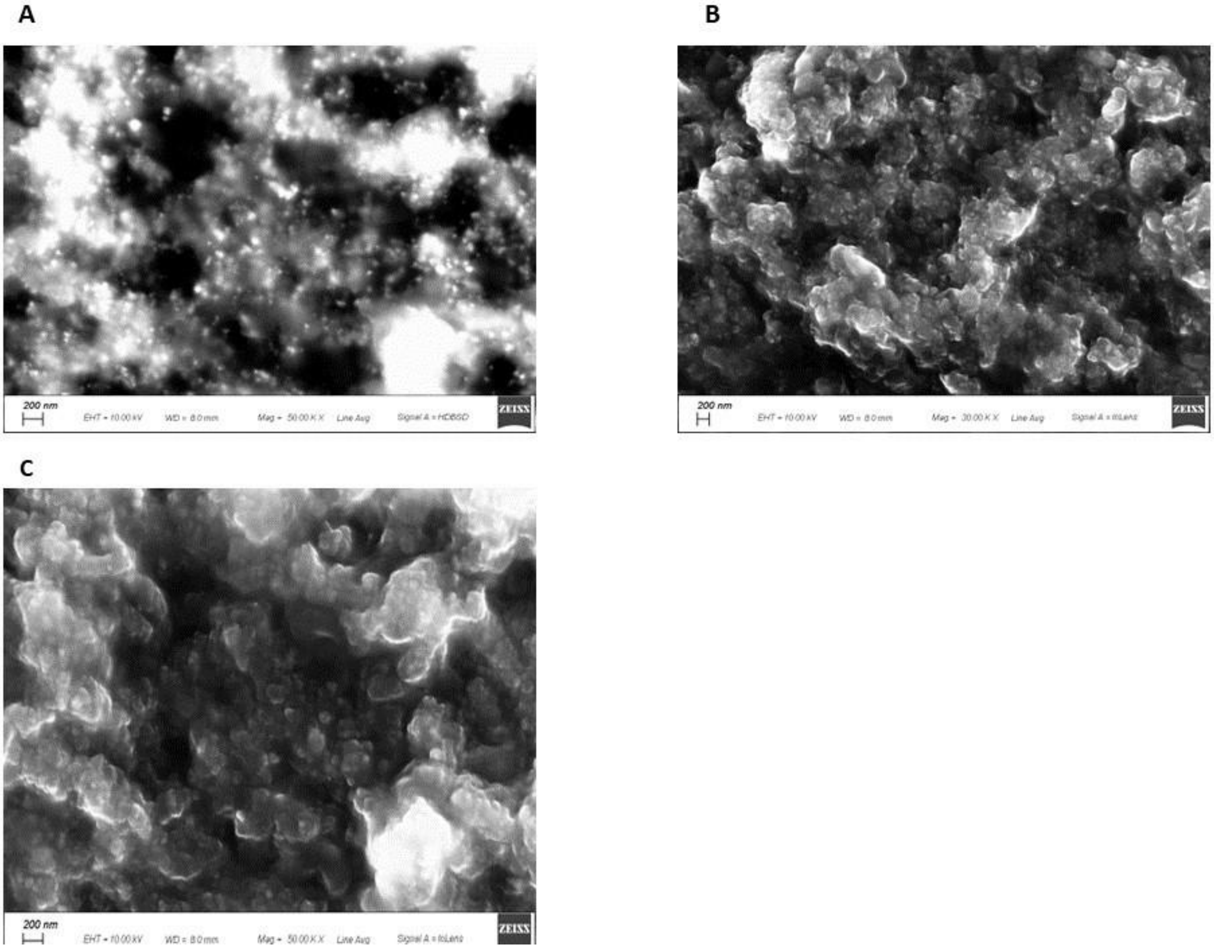

Figure S6. SEM images of AgNPs from *Rhodococcus* (A), *Brevundimonas* (B), and *Bacillus* (C).

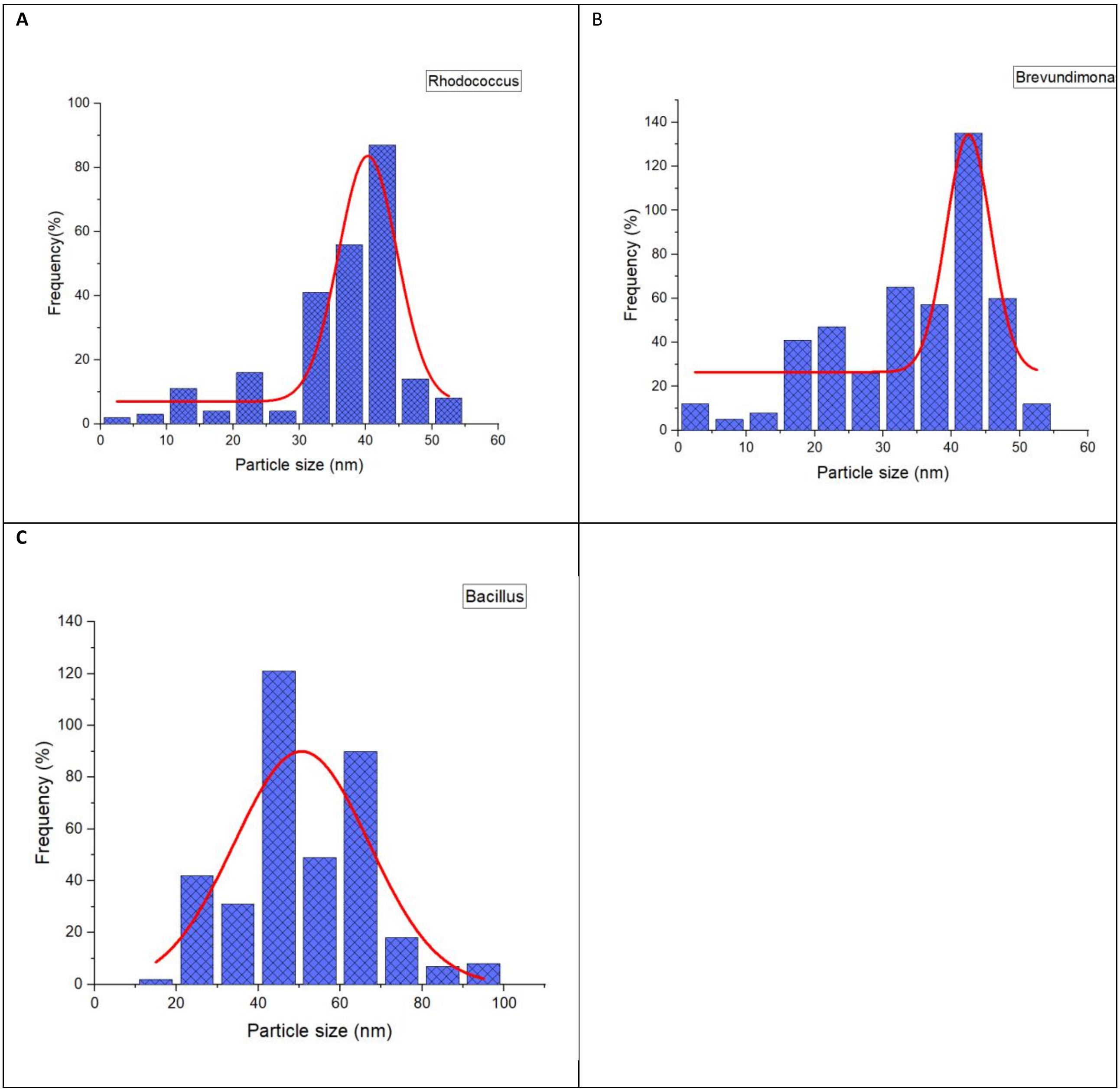

Figure S7. AgNPs average size from TEM distribution of *Rhodococcus* (A), *Brevundimonas* (B), and *Bacillus* (C).

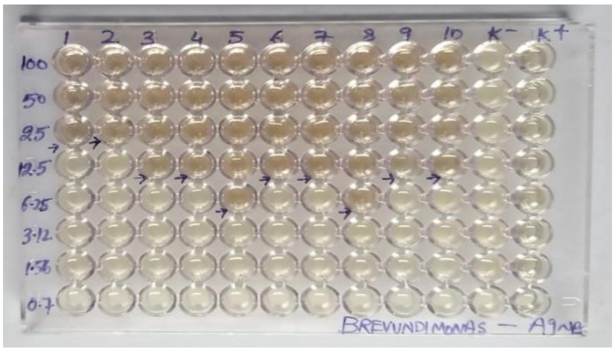

(**a**)

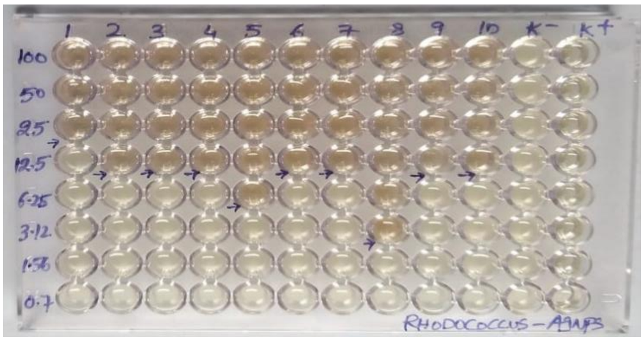

(**b**)

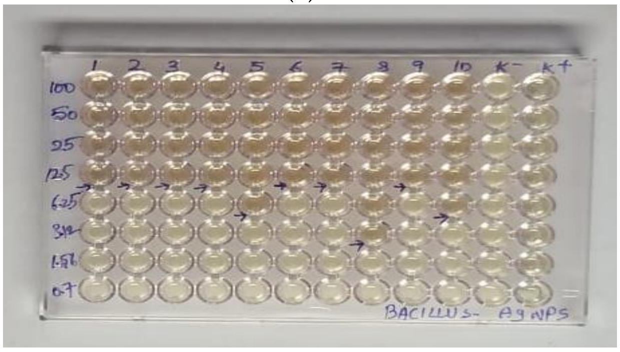

(**c**)

Figure S8. MIC assay of the Ag NPs synthesized by *Brevundimonas* (A), *Rhodococcus* (B) and *Bacillus* (C) by Broth microdilution method. 1. *Staphylococcus aureus* **2.** *Escherichia coli* **3.** *Klebsiella pneumoniae* **4.** *Pseudomonas aeruginosa* **5.** *Proteus mirabilis* **6.** *Citrobacter koseri* **7.** *Acinetobacter baumanii* **8.** *Serratia marcescens* **9.** *Candida albicans* **10.** *Candida parapsilosis*.
